# Colorectal Cancer Stem Cells Are Enriched in Xenogeneic Tumors Following Chemotherapy

**DOI:** 10.1371/journal.pone.0002428

**Published:** 2008-06-18

**Authors:** Scott J. Dylla, Lucia Beviglia, In-Kyung Park, Cecile Chartier, Janak Raval, Lucy Ngan, Kellie Pickell, Jorge Aguilar, Sasha Lazetic, Stephanie Smith-Berdan, Michael F. Clarke, Tim Hoey, John Lewicki, Austin L. Gurney

**Affiliations:** 1 OncoMed Pharmaceuticals Inc., Redwood City, California, United States of America; 2 Stanford Institute for Stem Cell Biology & Regenerative Medicine, Stanford University, Palo Alto, California, United States of America; Brigham and Women's Hospital, United States of America

## Abstract

**Background:**

Patients generally die of cancer after the failure of current therapies to eliminate residual disease. A subpopulation of tumor cells, termed cancer stem cells (CSC), appears uniquely able to fuel the growth of phenotypically and histologically diverse tumors. It has been proposed, therefore, that failure to effectively treat cancer may in part be due to preferential resistance of these CSC to chemotherapeutic agents. The subpopulation of human colorectal tumor cells with an ESA^+^CD44^+^ phenotype are uniquely responsible for tumorigenesis and have the capacity to generate heterogeneous tumors in a xenograft setting (*i.e.* CoCSC). We hypothesized that if non-tumorigenic cells are more susceptible to chemotherapeutic agents, then residual tumors might be expected to contain a higher frequency of CoCSC.

**Methods and Findings:**

Xenogeneic tumors initiated with CoCSC were allowed to reach ∼400 mm^3^, at which point mice were randomized and chemotherapeutic regimens involving cyclophosphamide or Irinotecan were initiated. Data from individual tumor phenotypic analysis and serial transplants performed in limiting dilution show that residual tumors are enriched for cells with the CoCSC phenotype and have increased tumorigenic cell frequency. Moreover, the inherent ability of residual CoCSC to generate tumors appears preserved. Aldehyde dehydrogenase 1 gene expression and enzymatic activity are elevated in CoCSC and using an *in vitro* culture system that maintains CoCSC as demonstrated by serial transplants and lentiviral marking of single cell-derived clones, we further show that ALDH1 enzymatic activity is a major mediator of resistance to cyclophosphamide: a classical chemotherapeutic agent.

**Conclusions:**

CoCSC are enriched in colon tumors following chemotherapy and remain capable of rapidly regenerating tumors from which they originated. By focusing on the biology of CoCSC, major resistance mechanisms to specific chemotherapeutic agents can be attributed to specific genes, thereby suggesting avenues for improving cancer therapy.

## Introduction

The presence of diverse cell populations in normal and neoplastic tissue has long been recognized. While normal tissue structure and function is facilitated by diverse cell types, generated during development and continually replaced to maintain homeostasis, cancer is generally characterized by disorganized overproliferation. Because genetic material is propagated over extended periods of time due to the self-renewal properties of stem cells, the compounding mutations required for tumorigenesis have been hypothesized to arise in these rare cells and not their more numerous progeny, which have a finite lifespan once committed to differentiation. Like normal tissue-resident stem cells that support the cellular hierarchy comprising a particular tissue over the lifespan of an individual, cancer stem cells (CSC) are defined by their ability to self-renew indefinitely, while maintaining their ability to generate both tumorigenic (TG) and non-tumorigenic (NTG) cells [1]. Unlike in normal development, however, neoplastic progenitor cell populations can gain self-renewal capabilities, thereby also fulfilling the definition of a CSC [2], [3]. Ultimately, demonstration of the self-renewal and differentiation capabilities that define a stem cell, both normal and neoplastic, can be confirmed by serial transplant studies that enable discrimination of cells possessing self-renewal ability versus those capable of numerous, though finite, non self-renewing cell divisions [4].

The CSC paradigm rests on the foundation that tumor heterogeneity can be generated by a single CSC. Because traditional cell lines and xenografts do not recapitulate the cellular and morphological heterogeneity observed in xenografts arising from implantation of tumor cells taken directly from patients and not passaged *in vitro*, CSC biology depends on the latter form of xenografts. Although the relevance of these, and all, xenografts to patient tumors has been questioned [5]–[7], transplantation of human cells into mice is the only model system that does not expose human cells to an aphysiological *ex vivo* environment comprised of tissue culture plastic and culture medium and which can recapitulate the cellular heterogeneity of a primary tumor *in vivo*. Another topic of controversy at the foundation of the CSC hypothesis is the proposition that complete heterogeneity of a tumor can result from a single cell [8], [9]. Disregarding the stromal, endothelial, and hematopoietic elements recruited and incorporated into the tumor, it has been suggested that TG and NTG populations arise from different cells and not from a single CSC [9]. If CSC can indeed generate the cellular heterogeneity observed in patient tumors, and mouse xenografts composed of cells strictly passaged *in vivo* best maintain characteristics of tumors in patients, then the focus of efforts in the cancer biology field should be squarely on these cells and their microenvironmental niche.

Chemotherapeutic strategies that target rapidly dividing cells have principally been used to treat tumors of epithelial origin. While often effective at debulking tumor mass, these agents have largely failed to eradicate disease [10]. A reason often attributed to this failure is that subsets of cells gain resistance to therapy through genetic mutation and natural selection. While this conjecture may hold true, particularly in a setting of prolonged treatment, one tenet of the “cancer stem cell hypothesis” posits that the cells responsible for tumor recurrence may inherently be more resistant to tumor debulking agents through any one of a number of mechanisms; thereby explaining refractory tumor growth following these treatments [11]. In support of this hypothesis, resistance to radiation can result from elevated expression of DNA damage response genes, as is the case for CD133^+^ glioblastoma stem cells [12]. In analogy to hematopoietic stem cells, solid tumor CSC have been proposed to exhibit high level expression of multidrug transporter family genes, such as ABCG2 and ABCB5, likely resulting in more efficient efflux of chemotherapeutic drugs [13]–[15]. CSC may also enter the cell cycle less frequently, allowing them to resist toxicity by drugs that target highly proliferative cells. Evidence for chemoresistance by stem-like cells in epithelial cell lines and xenogeneic tumor-derived cells has been presented [16]–[19]; however, these studies have either utilized cell lines adapted to tissue culture and/or do not assess altered tumor-initiating cell frequency *in vivo* following chemotherapy. By definition, tumor-initiating cells are enumerated retrospectively, therefore altered CSC frequencies post-therapy are best demonstrated by serial transplantation.

Cyclophosphamide (CPA) and Irinotecan are agents that target proliferating cells and are commonly used chemotherapeutic agents in the treatment of solid tumors. Through different mechanisms, both act to inhibit DNA replication resulting in the slowing or inhibition of cell division and resulting in apoptosis. Resistance to CPA has been suggested to result from high cytoplasmic aldehyde dehydrogenase (ALDH) enzyme activity: particularly that of ALDH1 and ALDH3 [20], [21], which oxidize and inactivate the bioactive metabolic byproduct of CPA, aldophosphamide/4-hydroxycyclophosphamide (4-HC) [22], [23]. ALDH1, in particular, may play a major role in CPA resistance, as it's *K_m_* for CPA is ∼52 µM, whereas that of other ALDH family members with CPA catabolic activity, ALDH3 (*ALDH3A1*) and SSDH (*ALDH5A1*), are 10-fold lower (*K_m_*>520 µM) [24]. Though many tissues are relatively resilient in the wake of CPA treatment [25], only hematopoietic and neural stem cells have been demonstrated to contain high ALDH activity in reconstituting transplantation studies following isolation of these cell populations [26]–[28]. Because colorectal cancer likely arises from colon stem or progenitor cells, it is tempting to hypothesize that similar mechanisms may render CoCSC resistant to CPA.

Cancer stem cell populations have now been prospectively identified from various tumors of epithelial origin, including the breast, colon and prostate [19], [29]–[33]. In all colorectal tumors we have investigated to date, tumors can be successfully transplanted using small numbers (*e.g.* <1,000) of cells phenotypically positive for both epithelial-specific antigen (*i.e.* ESA or EpCAM) and CD44 [30]. In some tumors, isolation of cells positive for CD166, in addition to ESA and CD44, further enriches for colorectal cancer stem cells (CoCSC), allowing for efficient tumorigenesis with as few as 200 ESA^+^CD44^+^CD166^+^ cells [30]. Not only does this phenotype identify CoCSC in xenograft tumors, but tumorigenesis can be initiated from primary tumor samples with a small number of ESA^+^CD44^+^CD166^+^ cells. The reproducible identification of CoCSC using ESA and CD44 facilitates not only therapeutic efficacy studies across all patient-derived xenografts studied to date, but also facilitates dissection of the molecular pathways involved in tumorigenesis and resistance to therapy.

Here we demonstrate that human ESA^+^CD44^+^ CoCSC generate adenocarcinomas that resemble parental tumors in both their phenotype and histology upon serial transplant in a xenograft setting and are enriched in tumors following classical chemotherapeutic regimens intended to shrink tumors. We further demonstrate both *in vitro* and *in vivo* that resistance to CPA is mediated, at least in part, by ALDH1 enzyme activity and that resistance to other chemotherapeutic agents (*e.g.* Irinotecan) is not likely attributed to this mechanism. To the best of our knowledge, this is the first functional demonstration that a human epithelial CSC population, as defined by serial transplantation, is enriched in residual solid tumors following chemotherapy. We further demonstrate that tumor heterogeneity in the form of a complex adenocarcinoma composed of TG and NTG populations can be generated by a single cell. Finally, the experimental methods used here provide a platform for assessing the efficacy of novel agents targeting solid tumor stem cells.

## Results

### CoCSC are enriched in residual tumors following CPA administration

**Figure 1:**
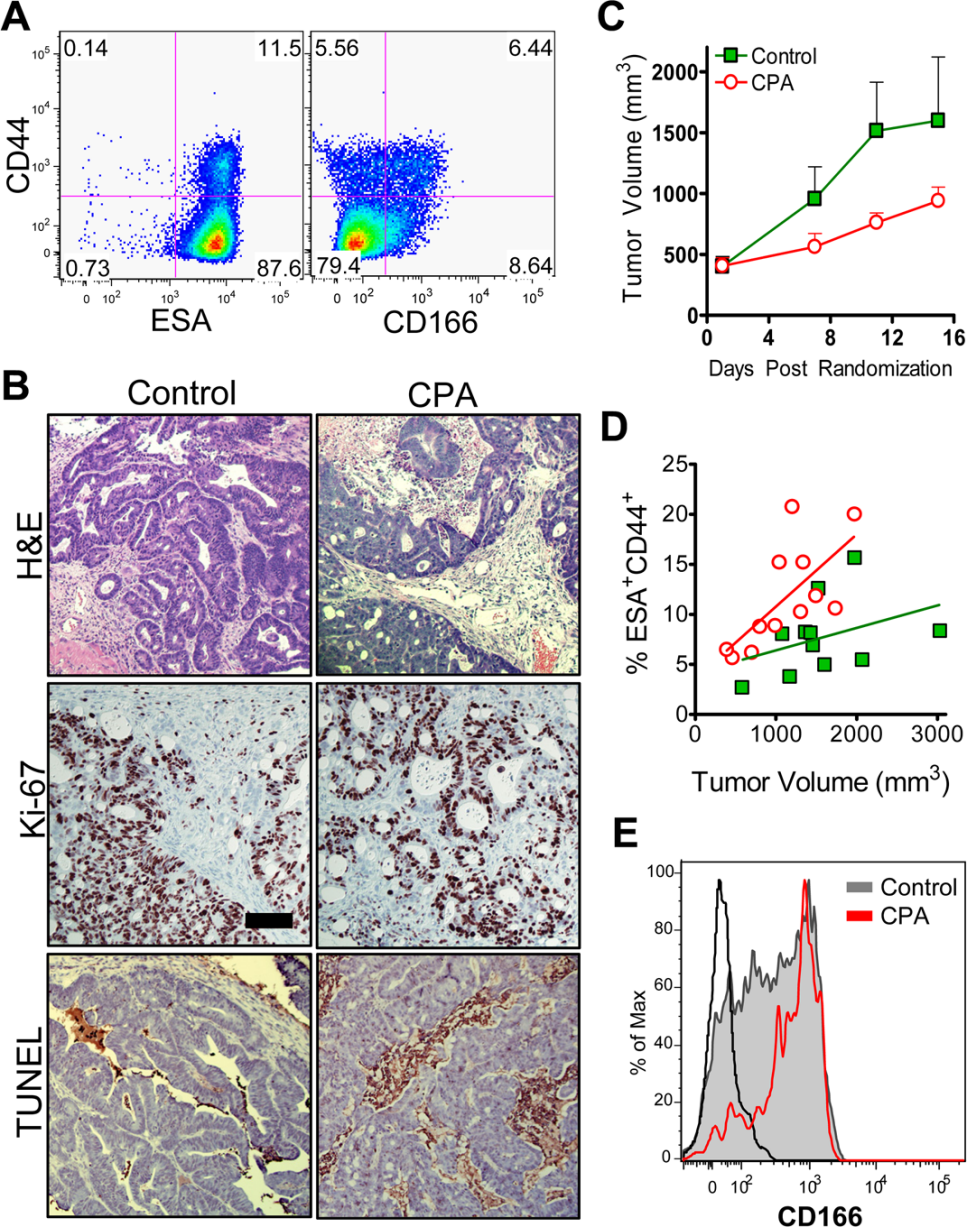
CoCSC phenotype cells preferentially survive CPA chemotherapy. *A*) Phenotypic profile of UM-C4 colorectal tumors for ESA, CD44 and CD166, following exclusion of mLin^−^ cells. *B*) Formalin fixed, paraffin embedded tumor sections from CPA- or vehicle-treated control mice stained with Hematoxylin & Eosin (H&E), for proliferating Ki-67^+^, or for TUNEL^+^ dead cells. Black bar = 100 µm. *C*) Following randomization to normalize treatment groups at 400 mm^3^ at day 0, twice weekly administration of vehicle (green boxes) or 38 mg/kg CPA (red circles) commenced and tumors were measured periodically. *D*) Phenotypic analysis of individual tumors, displaying the percentage of human tumor cells with the ESA^+^CD44^+^ phenotype as a function of tumor size. *E*) Representative overlay histogram displaying CD166 surface expression on human ESA^+^CD44^+^ tumor cells from vehicle- or CPA-treated animals. The black line represents isotype control staining of ESA^+^CD44^+^ tumor cells.

Early passage human xenograft tumors generated from cells never expanded *in vitro* appear to closely resemble those in patients and thus may serve as an excellent model by which to study cancer. We recently demonstrated that the subpopulation of human colorectal tumor cells with an ESA^+^CD44^+^ phenotype is uniquely responsible for generating heterogeneous adenocarcinomas in a xenograft setting (Figure 1A & B)[30]. Human ESA^+^CD44^+^ cells from xenogeneic colorectal tumors can be further subdivided based on CD166 expression, resulting in enrichment for tumorigenic (TG) cells among the CD166^+^ population. In contrast, all remaining cancer cells of human origin appear unable to initiate tumor growth and are referred to as non-tumorigenic (NTG). Having identified colorectal cancer stem cells (CoCSC) capable of fueling xenogeneic tumor growth, we sought to address a lingering question in the field regarding whether CSC are preferentially resistant to chemotherapeutic drugs.

Cyclophosphamide (CPA) is an alkylating agent whose metabolic byproduct, phosphoramide mustard, crosslinks DNA and induces apoptosis in rapidly dividing cells [22]. CPA is a commonly used chemotherapeutic drug in the treatment of various types of cancer, such as soft tissue sarcomas, breast cancer and non-Hodgkins lymphoma, but is not generally used to treat colorectal cancer due to a prevailing resistance on the part of these tumors. To investigate this resistance to therapy, we explored whether CoCSC are enriched in residual tumors following CPA administration *in vivo*. To address this subject of interest, tumors were initiated with highly purified CoCSC from multiple xenogeneic tumor lines (UM-C4 & UM-C6) and upon reaching ∼400 mm^3^, mice were randomized to receive either vehicle or 38 mg/kg CPA, twice weekly. Within 15 days of randomization and administration, tumor growth was noticeably retarded in the CPA-treated animals (Figures 1C and Figure S1A). Following euthanization, tumors were removed for histological and phenotypic analysis. Although the percentage of proliferating tumor cells (*i.e.* Ki-67^+^) did not appear altered, there was noticeably more cell death (i.e. TUNEL^+^ cells; Figure 1B) and ESA^+^CD44^+^ cells were more frequent in tumors from CPA-treated mice (Figure 1D). The trend towards more frequent ESA^+^CD44^+^ cells in CPA-treated tumors generally held true independent of tumor volume and was more pronounced at higher CPA doses (*e.g.* 38 mg/kg versus 25 mg/kg; data not shown). Enrichment of ESA^+^CD44^+^CD166^+^ cells was even more striking and particularly evident when CD166 expression was analyzed on residual human ESA^+^CD44^+^ cells (Figures 1E, S1B & C). Similar results were observed with both xenogeneic tumor lines investigated.

**Figure 2:**
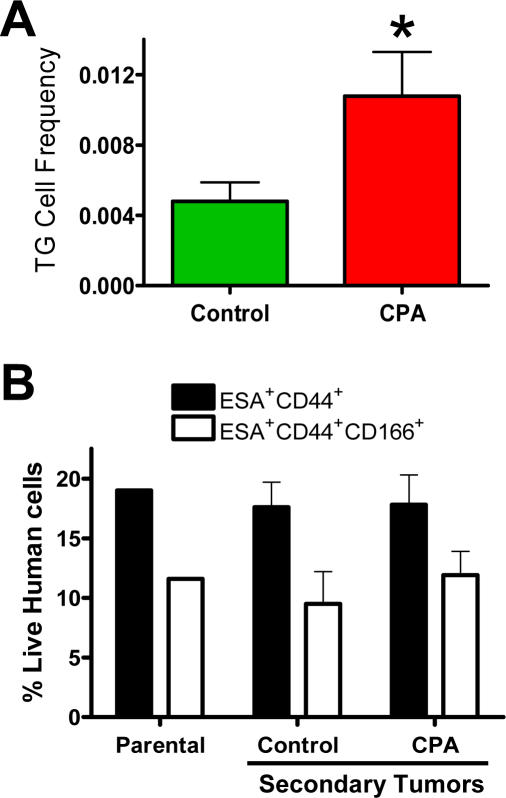
Tumorigenic UM-C4 cells are enriched in residual tumors following CPA administration. A) Limiting dilution analysis of unfractionated UM-C4 tumor cells was used to calculate TG cell frequency using *Poisson* distribution statistics (±95% confidence level; *P = 0.0016). *B*) Percentage of human ESA^+^CD44^+^ and ESA^+^CD44^+^CD166^+^ cells in secondary tumors arising from residual tumorigenic cells transplanted serially in limiting dilution following vehicle or CPA treatment regimens.

To determine whether the phenotypic increase in CoCSC following CPA administration correlated with an authentic rise in TG cell frequency, we serially transplanted 4 sets of mice with bulk UM-C4 tumor cells using a limiting dilution approach and scored them as positive or negative for tumor growth after three months. Mice with palpable tumors at 90 days were kept alive to determine whether tumor growth would continue beyond 200 mm^3^, as rare palpable masses can occur, but which contain murine stromal elements and no detectable human cells upon analysis at the termination of the study (data not shown). Based on *Poisson* distribution statistics, TG cells were >2.2-fold more frequent in CPA-treated tumors than in mice administered vehicle (*P* = 0.0016; Table 1 & Figure 2A). Importantly, the cellular phenotype of these serially transplanted secondary tumors were identical to the parental tumor cells used to initiate the study (Figure 2B), demonstrating that intervening treatment and serial transplantation of cells in limiting dilution did not alter either the tumorigenic potential of CoCSC nor their capacity to generate heterogeneous tumors containing predominantly non-tumorigenic (*i.e.* CD44^−^) cells.

**Table 1:** 

		Tumors/animals injected (%)			
Cell type injected	# cells	Control		CPA	
Bulk Tumor Cells	1500	18/18	(100%)	18/18	(100%)
	500	16/17	(94%)	19/19	(100%)
	167	11/18	(61%)	13/18	(72%)
	56	2/20	(10%)	12/20	(60%)
mLin^−^ESA^+^CD44^+^	50	3/6	(50%)	2/6	(33%)
mLin^−^ESA^+^CD44^+^CD166^+^	50	7/10	(70%)	8/10	(80%)

Because TG cells were more frequent and tumor cell populations from CPA-treated animals were enriched for phenotypes associated with CoCSC, we next isolated CoCSC phenotype cells from UM-C4 tumors of vehicle- versus CPA-treated mice and asked whether these cells inherently differed in their ability to generate secondary tumors. Serial transplantation of 50 ESA^+^CD44^+^ or ESA^+^CD44^+^CD166^+^ cells from tumors in CPA- versus vehicle-treated control mice resulted in secondary tumors with roughly equal frequency (Table 1), which when considered in conjunction with previous observations suggest that CoCSC are more frequent within tumors exposed to CPA chemotherapeutic regimens and are unaffected in their ability to fuel tumor growth.

### CoCSC have high expression of *ALDH1A1*

**Figure 3:**
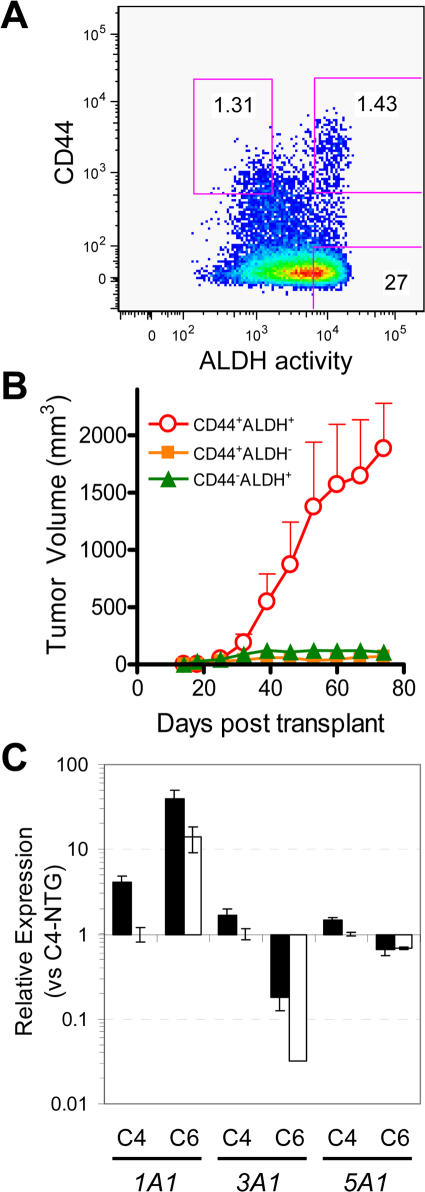
ALDH1 enzyme activity demarcates a subpopulation of CoCSC. *A*) Phenotypic profile of human ESA^+^ UM-C4 tumor cells for ALDH1 enzymatic activity. Gates demarcate tumor subpopulations isolated by FACS for tumorigenicity studies, wherein *B*) growth kinetics of CD44^+^ALDH^+^ (red circles), CD44^+^ALDH^−^ (orange boxes) and CD44^−^ALDH^+^ (green triangles) populations are plotted. Measurements reflect only mice with palpable tumors. Data representative of n = 3 independent experiments. *C*) Taqman qRT-PCR data displaying relative expression of *ALDH1A1* (*1A1*), *ALDH3A1* (*3A1*) *and ALDH5A1* (*5A1*) in tumorigenic (TG; black) versus non-tumorigenic (NTG; white) cells (n≥2) from 2 patient-derived xenogeneic colorectal tumor lines (UM-C4 & UM-C6). Data reflects Mean±SEM, is normalized versus *GUSB* and displayed relative to UM-C4 NTG expression for each gene.

In searching for additional CoCSC markers, we previously used a tool that can identify hematopoietic and neural stem cell populations: ALDH enzyme activity [30]. Using the Aldefluor™ reagent, which undergoes a shift in fluorescence following enzymatic cleavage by ALDH enzymes, and the ALDH1-specific inhibitor diethylaminobenzaldehyde (DEAB) [34], a large subpopulation of ESA^+^CD44^+^ cells from both UM-C4 and UM-C6 tumor lines was determined to have high ALDH activity (Figure 3A) [30]. Similar observations were also made in other patient-derived xenograft colorectal tumor lines (Figure S2). Furthermore, when ESA^+^CD44^+^ cells were subdivided based on ALDH activity and isolated by FACS, ALDH^+^ cells were tumorigenic in all cases investigated [30]. Of note, tumorigenicity is strictly conferred by ESA^+^CD44^+^ALDH^+^ cells in the UM-C4 colorectal tumor line (Figure 3B). Although tumors can arise from ESA^+^CD44^+^ALDH^−^ cells taken from the UM-C6, OMP-C5 and OMP-C8 tumor lines [30], an ALDH^+^ subset of TG ESA^+^CD44^+^ cells does exist in all xenogeneic colorectal tumor lines investigated to date (n = 6). In contrast, a subpopulation of CD44^−^ cells with high ALDH activity also exists in all tumor lines, but these cells fail to generate tumors upon transplantation.

A subset of ALDH enzymes can oxidize, and thus inactivate, the cytotoxic CPA metabolite 4-HC/aldophosphamide [23], [35]. The presence of cytoplasmic ALDH1 and ALDH3, in particular, have been associated with CPA resistance in the A549 lung cancer cell line [20], [21]. ALDH1 (encoded by *ALDH1A1*) is greater than 10-fold more efficient at catabolizing 4-HC/aldophosphamide than its related family members ALDH3 and SSDH: encoded by the *ALDH3A1* and *ALDH5A1* genes, respectively [24]. In Taqman™ qRT-PCR analysis of UM-C4 and UM-C6 tumors, *ALDH1A1* is generally expressed at higher levels than *ALDH3A1* and *ALDH5A1*. *ALDH1A1* gene expression is 2.8-fold higher in CoCSC than in NTG cells, and despite slight elevation of *ALDH3A1* expression in CoCSC versus NTG populations (Figure 3C), its message is barely detectible in either tumor line. Additionally, whereas *ALDH5A1* is slightly elevated in TG versus NTG cells from UM-C4 tumors (∼1.5-fold), its expression is similar among these populations from UM-C6 tumors (Figure 3C). The preferential expression of *ALDH1A1* in TG cells supports observed enzymatic activity measurements among the human ESA^+^CD44^+^ subpopulation, wherein the majority of these cells have high DEAB-sensitive, ALDH1 activity. When considered in conjunction with the 10-fold higher proficiency of ALDH1 versus ALDH3 and SSDH at inactivating CPA intermediates [24], one might interpret these results to suggest that CoCSC resistance to CPA might be predominantly mediated by ALDH1 enzymatic activity.

### *ALDH1A1*, *MYC*, and *MYB* are enriched in residual TG cells post CPA therapy

**Figure 4:**
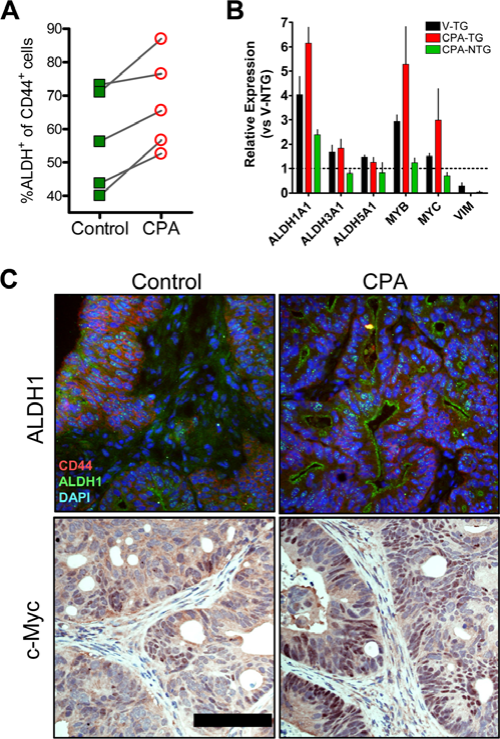
CoCSC with high ALDH1 activity are more prevalent following CPA therapy. *A*) Ratio of ALDH^+^ tumor cells among the human ESA^+^CD44^+^ population in tumors from vehicle-treated control or CPA-treated mice. Data reflects the paired Mean of 5 independent experiments using 3 different xenogeneic colorectal tumor lines (UM-C4, UM-C6 & OMP-C8) and n≥5 mice per experiment. *B*) Taqman qRT-PCR data for the denoted genes using TG and NTG populations isolated from (V) vehicle- or CPA-treated UM-C4 tumors. Data represents Mean±SEM (n≥2). *C*) Immunofluorescence or immunoperoxidase staining of frozen or formalin-fixed, paraffin embedded tumors for ALDH1 or c-Myc, respectively, from vehicle- and CPA-treated mice. Black bar = 100 µm.

Since *ALDH1A1* gene expression is elevated in TG versus NTG cells, ALDH1 enzymatic activity is disproportionably high in cells with the CoCSC phenotype, and this activity may mediate resistance to CPA, we next asked whether the frequency of ESA^+^CD44^+^ALDH^+^ cells was elevated in UM-C4, UM-C6 and OMP-C8 tumors from mice receiving CPA versus those being administered vehicle. Like the observed phenotypic increase in CD166^+^ cells (Figures 1E, S1B & S1C), the ALDH^+^ subpopulation of ESA^+^CD44^+^ cells was consistently higher in CPA- versus vehicle-treated control mice (67.6±6.3% versus 56.8±6.8%, respectively; n = 5, *P*<0.012 in paired *t*-test) (Figure 4A).

A number of gene products have been deemed critical to normal colon development and are widely expressed in colorectal tumors [36]–[39]. Following the isolation of TG versus NTG populations from both vehicle- and CPA-treated UM-C4 tumors with >99% purity, expression of numerous genes with suggested ties to colorectal cancer was determined by Taqman™ qRT-PCR. Those differentially expressed in TG versus NTG cells included not only *ALDH1A1*, as described above (Figure 3C), but also c-Myb (*MYB*) and c-Myc (*MYC*; Figure 4B). In contrast, genes associated with differentiation and a mesenchymal phenotype [40], [41], such as Vimentin (*VIM*), were more highly expressed in NTG cells.

Consistent with the elevated frequency of residual tumor cells expressing high levels of tumorigenicity-associated markers, such as CD166, relative expression of CoCSC-associated versus NTG-associated transcripts, such as *ALDH1A1* and *VIM*, respectively, was further disparate in CPA- versus vehicle-treated control UM-C4 tumors (Figure 4B). Consistent with the hypothesis that ALDH1 may have a role in mediating resistance to CPA, *ALDH1A1* gene expression was further elevated in residual cells with the CoCSC phenotype, but *ALDH3A1* and *ALDH5A1* were not (Figures 4B & S3). In addition, genes previously identified as highly expressed in the epithelial stem/progenitor cell compartment at the base of colon crypts and in neoplastic tissue (*e.g. MYB* & *MYC*) were more highly expressed in residual tumor CoCSC than the NTG population of cells that represent the bulk of the tumor, but which are generated by CoCSC and appear more susceptible to CPA-induced cytotoxicity. Similar expression patterns were also observed in other xenogeneic tumor lines following CPA-treatment, including UM-C6 (Figure S3).

Finally, gene expression differences in CPA- versus vehicle-treated control tumors were further correlated with protein expression by immunofluorescence and IHC. Intracellular levels of ALDH1 and nuclear c-Myc, for example, were more prevalent in CPA-treated tumors (Figure 4C). Interestingly, ALDH1 staining in CPA-treated tumors appeared to localize to the apical surface, where it may be hypothesized to intercept CPA metabolites as they enter the cell. In contrast, ALDH1 protein levels were lower and more diffuse in control tumors. Similarly, c-Myc was noticeably more concentrated in the nucleus of colorectal tumor cells of CPA-treated mice, consistent with the increased frequency of TG cells and elevated *MYC* expression within this resilient population.

### CoCSC resistance to chemotherapy is not specific to CPA

**Figure 5:**
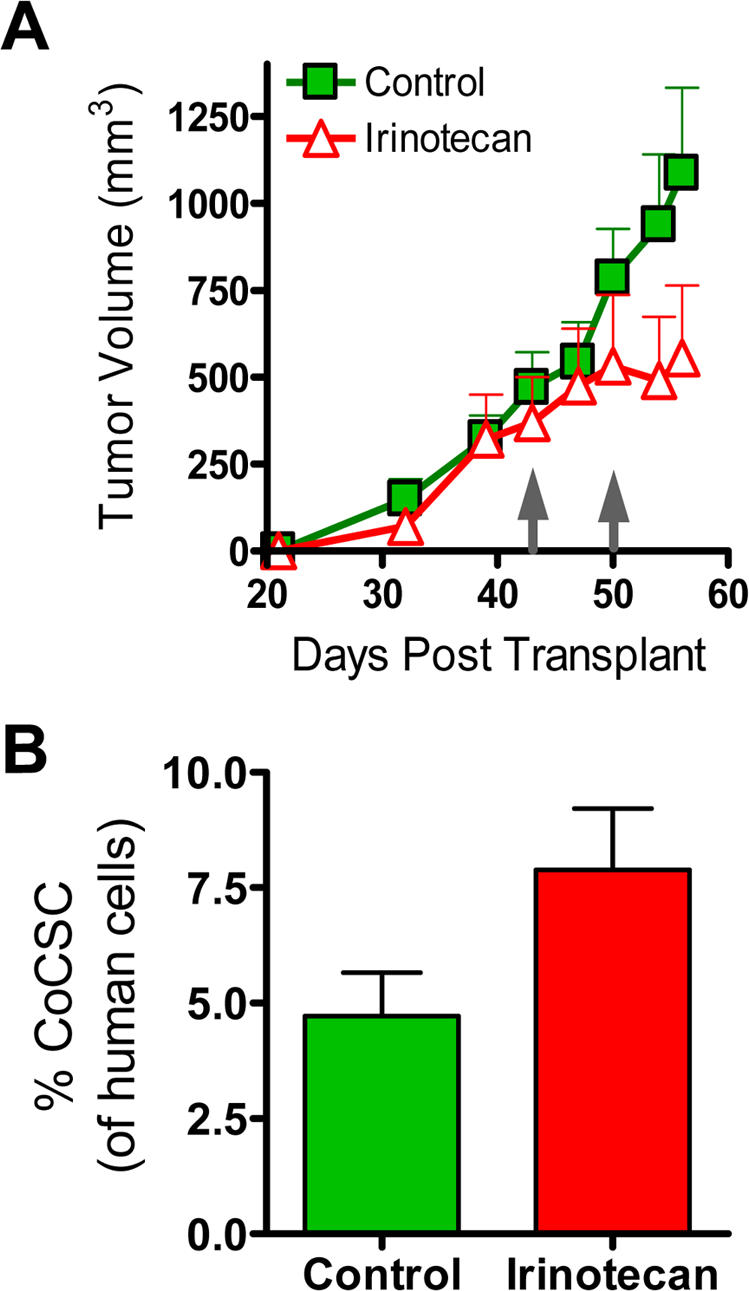
CoCSC phenotype cells are also enriched following Irinotecan treatment. *A*) Upon randomization to normalize treatment groups at 400 mm^3^ at day 43, once weekly administration of vehicle (green boxes) or 15 mg/kg Irinotecan (red triangles) commenced and tumors were measured twice weekly. Growth curves representing the Mean±SEM are shown. B) Percentage of human ESA^+^CD44^+^CD166^+^ CoCSC phenotype cells in residual tumors is shown. Data reflects n≥4 mice per treatment group.

To determine whether CoCSC also exhibit resistance to other important chemotherapeutic agents, we examined the impact of Irinotecan (*a.k.a.* SN-38) on CoCSC frequency. Experiments were initiated whereupon tumor bearing mice received weekly injections of 15 mg/kg Irinotecan. After a slight delay of approximately 1 week, tumor growth was halted by Irinotecan (Figure 5A). Upon tumor harvest two weeks after the initiation of chemotherapy, the percentage of remaining CoCSC was assessed by flow cytometry. In Irinotecan-treated tumors, the percentage of bulk tumor cells with the CoCSC phenotype was increased 61% versus control mice administered vehicle (Figure 5B). Next, the inherent tumorigenicity of ESA^+^CD44^+^CD166^+^ cells from vehicle- or Irinotecan-treated mice was tested in a serial transplant setting versus those cells that did not phenotypically classify as CoCSC. Similar take rates were observed with CoCSC from control- or Irinotecan-treated tumors in these transplants, and cells phenotypically classified as “Other” did not initiate tumors (data not shown). The increased frequency of CoCSC phenotype cells in residual tumors from mice administered Irinotecan and the inability of cells with other phenotypes to transplant disease, together suggest that CoCSC are also preferentially resistant to Irinotecan.

### CPA resistance is specific to cells with high ALDH1 activity

**Figure 6:**
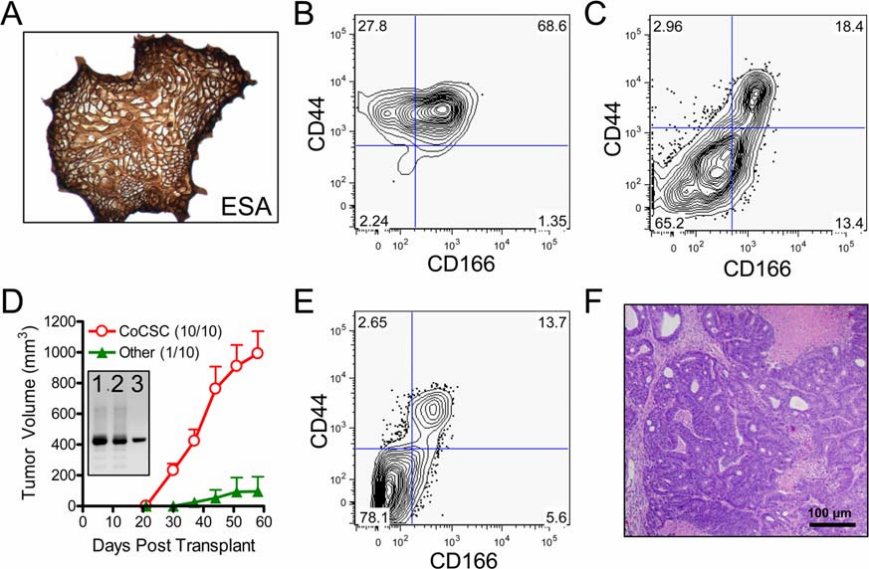
*In vitro* maintenance and expansion of CoCSC. Human ESA^+^CD44^+^CD166^+^ cells were plated in limiting dilution and cultured for fourteen days in serum-free maintenance conditions. Colorectal tumor colonies were then either analyzed for *A*) ESA expression by IHC, or *B*) ESA, CD44 and CD166 expression by flow cytometry. *C*) Cellular phenotype of single colony-derived tumors, showing human ESA^+^ cell subpopulations expressing CD44 and CD166. *D*) Tumor growth curves are shown for either 2,000 CoCSC phenotype cells or an equal number of cells with all other phenotypes (Other), which were isolated from *in vitro* colony-derived tumors (tumors/animals injected). Inset shows lentiviral insertion band obtained by inverse PCR of *1*) human xenograft tumor cells, *2*) ESA^+^CD44^+^CD166^+^ (CoCSC) cells or *3*) ESA^+^CD44^−^ (Other) cells isolated by FACS. Phenotypic and morphological analysis of single-cell derived tumors from serially transplanted CoCSC show that the diverse *E*) phenotype and *F*) histological makeup of xenogeneic colorectal tumors are maintained following brief *in vitro* culture in limiting dilution. Black bar = 100 µm.

Seeing that ESA^+^CD44^+^ tumor cells have high ALDH activity, the frequency of ESA^+^CD44^+^ALDH^+^ cells is increased in tumors from mice treated with CPA, and *ALDH1A1* gene expression is elevated in CPA-resistant CoCSC, we therefore sought to determine whether resistance to CPA was mediated by ALDH1 enzyme activity. Because the ALDH1 specific inhibitor DEAB is highly unstable *in vivo* [42], *in vitro* culture conditions that support TG colorectal tumor cell expansion were established. Notably, the cellular phenotype of cells best able to establish colonies *in vitro* was ESA^+^CD44^+^CD166^+^ (also that of CoCSC), whereas FACS purified CD44^−^ cells were unable to do so (data now shown). To validate that this culture system supports maintenance of TG cells, we sorted ESA^+^CD44^+^CD166^+^ CoCSC in limiting dilution into plates containing Mitomycin C-treated 3T3 or MEF feeder cells as support, depending on the tumor line used: UM-C4 or UM-C6, respectively. Colony formation frequency of CoCSC under these conditions was roughly similar to the TG cell frequency observed upon transplantation (∼1∶86±33; n = 7 for UM-C4 and 1∶52±5; n = 3 for UM-C6). Colonies were cultured for 14 days without passaging prior to reimplantation into mice. Not only did cells maintain ESA, CD44 and CD166 expression during *in vitro* culture (Figures 6A & B), but the ability to generate heterogeneous tumors resembling parental tumors was conserved as well (Figure 6C). To validate that tumor-initiating cells in tumors originating from single *in vitro* colonies both resembled parental tumors and were derived from a single cell, UM-C4 cells were transduced with a lentivirus carrying GFP-Luciferase, tumors were generated, and single colonies obtained from ESA^+^CD44^+^CD166^+^ cells were transplanted into mice. Upon harvest of these tumors derived from a single colony, either cells with the CoCSC phenotype or all other tumor cells with differing phenotypes were isolated by FACS, and tumorigenicity and clonality studies were performed. An input of 2,000 FACS purified cells resulted in a 100% take frequency with CoCSC phenotype cells, whereas human cells with all other phenotypes generated only 1 tumor (of 10 mice transplanted); likely resulting from rare contaminating CoCSC (Figure 6D). Of note, tumor heterogeneity can result from a single cell, as lentiviral transduction studies show that both ESA^+^CD44^−^ (*i.e.* NTG) and ESA^+^CD44^+^CD166^+^ (*i.e.* CoCSC) cells have identical lentiviral insertion sites (Figure 6D inset), but only CoCSC are able to propagate tumors. Phenotypic and histological analysis of these serially transplanted tumors, which originated from single *in vitro* colonies, show that the ability to generate adenocarcinomas similar to parental UM-C4 tumors is maintained during short-term *in vitro* culture in these conditions (Figures 6E & F).

**Figure 7:**
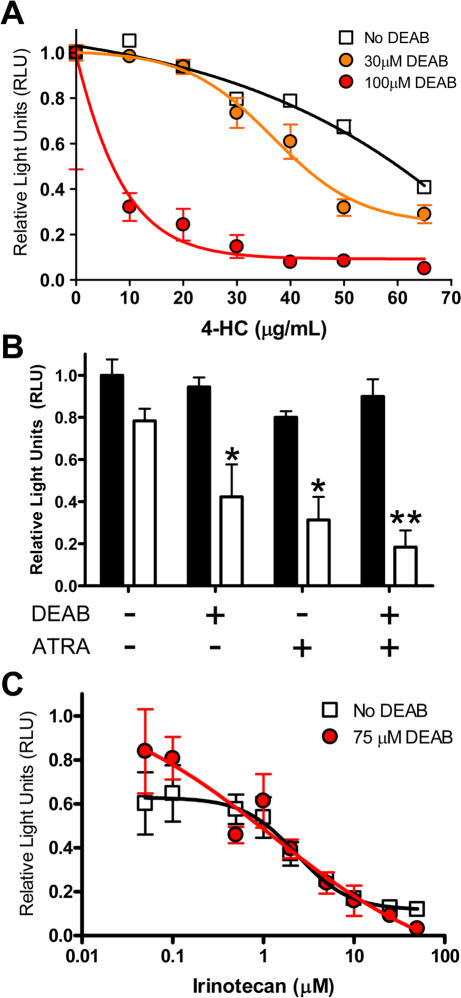
ALDH1 enzyme inhibition sensitizes colorectal tumor cells to CPA in vitro. *In vitro* cell viability measurements of human colorectal tumor cells following 4 hours of exposure to *A*) varying concentrations of 4-HC and/or the ALDH1-specific inhibitor DEAB, or *B*) 20 µg/mL 4-HC in the presence or absence of 75 µM DEAB and/or 1 µM ATRA. C) Cell viability measurements following 7 days of exposure to varying concentrations of Irinotecan in the presence or absence of 75 µM DEAB. All data is expressed as the Mean±SEM of triplicate measurements and is normalized versus vehicle-treated controls. All data is representative of n≥2 independent experiments using either UM-C4 or UM-C6 tumor cells. *P<0.015. **P = 0.003.

To test whether CPA resistance is mediated by ALDH1 enzyme activity, UM-C4 or UM-C6 colorectal tumor cell colonies established *in vitro* over the course of 3–4 days were then exposed to the bioactive CPA-metabolite, 4-HC, for 4 hours without or including various concentrations of the ALDH1-specific inhibitor, DEAB. In the absence of DEAB, the EC_50_ of 4-HC was ∼60 µg/mL (Figure 7A). Inhibition of ALDH1 activity resulted in increased susceptibility to 4-HC mediated cell death, as the EC_50_ was shifted to 43 µg/mL and 6 µg/mL in the presence of 30 µM and 100 µM DEAB, respectively. Like DEAB, all-trans retinoic acid (ATRA) has been demonstrated to reduce ALDH enzyme activity; however, it does so by reducing ALDH1 and ALDH3 protein levels [43]. Like DEAB, pre-treatment with 1 µM ATRA sensitized colorectal tumor colonies to 4-HC (n = 3; *P*<0.015) (Figure 7B). Surprisingly, the combination of ATRA pre-treatment and concurrent DEAB exposure synergized to facilitate increased chemosensitivity (n = 3; *P* = 0.003). Preferential *ALDH1A1* gene expression in CoCSC and sensitization of colorectal tumor cells to 4-HC by inhibiting ALDH1, together strongly suggest that ALDH1 enzyme activity mediates CPA resistance.

The above observation raised the question as to whether high ALDH1 enzyme activity also confers resistance to Irinotecan. We next established colonies of colorectal tumor cells and exposed them to serial dilutions of Irinotecan for one week in the presence or absence of 75 µM DEAB; a concentration known to potently inhibit ALDH1 activity. In contrast to 4-HC, the unaltered cytotoxicity profile of Irinotecan despite the presence of DEAB (Figure 7C) suggests that inherent resistance of CoCSC to Irinotecan occurs by a mechanism other than ALDH1 enzyme activity. Of note, neither DEAB nor an equal volume of its vehicle (1∶200 dilution of 95% Ethanol) significantly reduced proliferation or survival during extended culture (7 days), suggesting that ALDH1 activity is only critical in a chemotherapeutic setting.

### ALDH1 is a major mediator of CoCSC resistance to CPA

**Figure 8:**
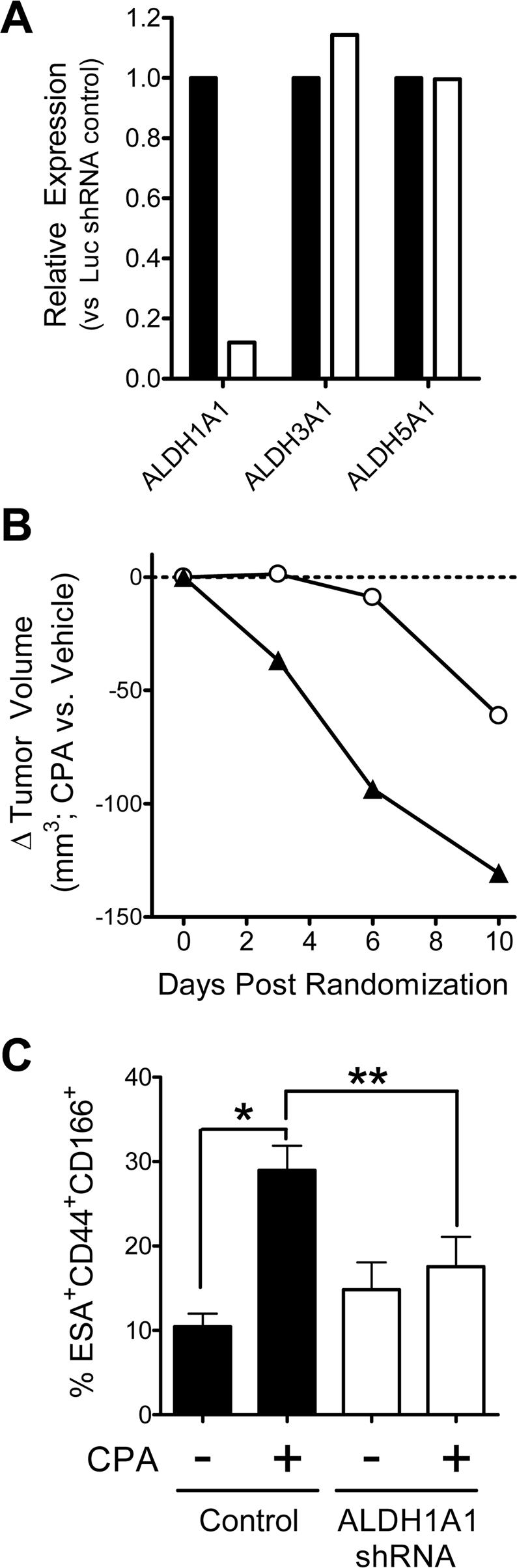
Knockdown of ALDH1A1 gene expression sensitizes tumors to CPA in vivo. UM-C6 tumor cells transduced with Luciferase- (black) or *ALDH1A1*-targeted shRNA (white) were *A*) assessed by Taqman™ qRT-PCR for relative expression of *ALDH1A1*, *ALDH3A1* and *ALDH5A1*, or *B*) transplanted into mice at 400 cells/mouse to initiate tumors. Following randomization when tumors reached a Mean volume of 175 mm^3^, mice were treated twice weekly with either vehicle or 38 mg/kg CPA. The Mean difference in tumor volume between CPA- and vehicle-treated mice is plotted for Luciferase shRNA control (open circles) or ALDH1A1 shRNA-containing (triangles) cells. *C*) Percentage of human ESA^+^CD44^+^CD166^+^ CoCSC phenotype cells in residual tumors is shown. Data reflects n≥5 mice per treatment group.

The above *in vitro* studies strongly suggest that the *ALDH1A1* gene product, ALDH1, mediates resistance to CPA. To determine the importance of ALDH1 to CoCSC tumorigenicity and resistance to CPA, we transduced UM-C6 tumor cells with shRNA targeting *ALDH1A1*. UM-C6 tumors were chosen because of their high *ALDH1A1* expression and relatively low expression of other ALDH family members (*e.g. ALDH3A1* & *ALDH5A1*; see Figure 4C). Cells successfully transduced with either an *ALDH1A1*-targeted shRNA or Luciferase-targeted shRNA control vector were isolated by FACS and implanted subcutaneously to generate tumors. Taqman™ qRT-PCR studies of UM-C6 tumor cells verified that *ALDH1A1* gene expression was reduced 88% versus Luciferase-targeted shRNA transduced control cells, whereas neither *ALDH3A1* nor *ALDH5A1* gene expression were altered (Figure 8A). After tumors reached roughly 175 mm^3^, tumors were randomized within each group and then either treated with vehicle or 38 mg/kg of CPA, twice weekly. By ten days post-randomization, tumors in vehicle-treated mice from both groups had almost doubled in size. Of note, the growth of *ALDH1A1*-targeted shRNA transduced cells did not differ significantly from controls (not shown). Consistent with observations described above, control Luciferase-targeted shRNA transduced tumors undergoing a regimen of CPA were retarded in their growth versus vehicle-treated controls (Figure 8B; open circles). In agreement with results observed *in vitro* with the ALDH1 inhibitor, DEAB, shRNA targeting of *ALDH1A1* gene expression appeared to sensitize tumors to CPA *in vivo*, as tumor growth essentially stopped, whereas *ALDH1A1*-targeted shRNA cells treated with vehicle continued to grow (Figure 8B; black triangles). Compared to vehicle-treated, *ALDH1A1*-targeted shRNA containing tumors, the difference in tumor volumes between CPA and vehicle-treated groups significantly differed by day 10 (n≥5, *P* = 0.0061).

Upon termination of the study, tumors were removed and the cellular phenotype was assessed to determine whether CoCSC frequency differed in tumors where *ALDH1A1* gene expression was being suppressed by shRNA. Whereas control Luciferase-targeted shRNA tumors were significantly enriched for ESA^+^CD44^+^CD166^+^ cells in CPA- versus vehicle-treated mice (n≥5, *P = 0.0002), both CoCSC and NTG cells from *ALDH1A1*-targeted shRNA containing tumor cells appeared equally sensitive to CPA, as the frequency of CoCSC was no different (Figure 8C). Of note, shRNA-mediated knockdown of ALDH1A1 *in vivo*, significantly reduced the frequency of CoCSC in residual tumors following CPA therapy (n≥6, **P = 0.045).

## Discussion

While recent decades have witnessed a revolution in therapeutic strategies yielding significant clinical responses measured in terms of tumor regression and disease-free survival, overall survival has failed to substantially improve. The recent identification of tumor cell subpopulations with the unique ability to fuel tumor growth (*i.e.* CSC) may shed light on the disconnect between response rates and overall survival. That is, therapies that fail to adequately target CSC populations, which represent a minority of most epithelial tumors, will fail to eliminate those cells capable of regenerating the tumor after therapy has ceased. Furthermore, survival of these long-lived cells in the presence of toxic therapeutic agents provides an ideal selective vice for additional mutations. Familiarity with both the means of resistance to a particular chemotherapeutic agent and the phenotypic identity of those cells that harbor resistance mechanisms should help facilitate the discovery of therapies better able to clear minimum residual disease and prolong overall survival. Although the cancer stem cell paradigm explains tumor heterogeneity, provides rationale for how genetic mutations might be accumulated over long time periods and suggests resistance to chemotherapeutics/radiation may be inherent and not acquired properties of specific tumor cell subpopulations in epithelial tumors [1], [2], a few key tenets of this theory have not been supported experimentally. Having previously identified surface markers that reliably identify colorectal cancer stem cells (CoCSC)[30], we show here that colorectal tumors are enriched for CSC following chemotherapeutic regimens that halt, or at least slow, tumor growth. ALDH1 enzymatic activity, which is generally highest in CoCSC, appears to play a major role in mediating resistance to CPA, as its inhibition *in vitro*, and reduced expression *in vivo*, sensitizes colorectal tumor cells to the bioactive metabolite of CPA. This chemotherapeutic resistance mechanism does not appear universal; however, as altered cytotoxicity profiles were not observed with other chemotherapeutic agents (*e.g.* Irinotecan) *en lieu* of ALDH1 inhibition.

ESA and CD44 demarcate the subpopulation of cells with tumorigenic ability in all colorectal tumors examined to date. Overexpressed in a number of epithelial tumors and suggested to be an important prognostic marker of tumor progression [44], CD166 (*i.e.* ALCAM) appears to further segregate TG from NTG cells when used in combination with ESA and CD44 [30]. Consistent with the hypothesis that TG cells are more resistant to chemotherapy and the association between CD166 expression and poor outcome, the tumorigenic CD166^+^ subset of ESA^+^CD44^+^ cells appeared more resilient to not only CPA, but also Irinotecan.

Like the normal colon crypt, which is predominantly composed of two different cell lineages (*i.e.* absorptive colonocytes and goblet cells), colorectal adenocarcinomas appear to contain both immature and mature colorectal cell lineages that are somewhat unstructured in their organization. Proto-oncogenes such as c-Myb not only coordinate normal development of the distal colon, but altered expression is commonly associated with hyperproliferation of immature colorectal cells and overt cancer [37], [45]. c-Myc has recently been demonstrated to mediate nuclear β-catenin-mediated tumorigenesis in the APC-deficient mouse model of intestinal neoplasia [46]. In fact, *MYB* appears to be a downstream target of c-Myc. Of significant interest in studies performed here is the observation that *MYC* expression is elevated in TG versus NTG cells from xenogeneic colorectal tumors, and that *MYC* levels are further increased in residual cells with the CoCSC phenotype. Conversely, *MYC* levels do not change, or are reduced in the NTG contingent of tumor cells, which themselves are progeny of CoCSC. These observations corroborate past studies demonstrating the association of the *MYB* and *MYC* proto-oncogenes with cancer, but underscore the distinction between TG versus NTG cells, in that those cells most resistant to therapy (*i.e.* CoCSC) also most resemble stem/progenitor cells in their phenotype and gene expression profiles.

Establishing mechanisms of resistance to chemotherapeutic drugs can be difficult, especially with heterogeneous xenogeneic tumors. However, *in vitro* culture conditions that facilitate colony formation with an input of tumorigenic colorectal tumor cells have been established and offer a new approach to the characterization of underlying mechanisms of drug resistance. Culture of CoCSC in these conditions is herein demonstrated to generate morphologically and histologically diverse tumors from limiting dilutions of colorectal tumor cells. That is, minimally cultured individual colonies generated *in vitro* are able to generate tumors *in vivo* that resemble the parental adenocarcinomas from which they were obtained. We further show for the first time that tumor heterogeneity can result from a single CoCSC using classical lentiviral insertion site analysis. Because *in vitro* colonies are highly enriched for TG ESA^+^CD44^+^CD166^+^ cells, the fate of these cells in defined culture conditions can now be assessed without the dangers encompassing extended *in vitro* culture and passaging.

Intracellular ALDH enzymes oxidize aldehydes to carboxylic acids and carry out various catabolic processes, including ethanol and amine catabolism and conversion of vitamin A to retinoic acid [23]. ALDH enzyme activity can also protect cells from the cytotoxic affects of CPA, as a subset of ALDH enzyme family members can catabolize the bioactive metabolite of CPA, aldophosphamide/4-HC [22]. Although *ALDH1A1*, *ALDH3A1*, and *ALDH5A1* gene products can degrade biologically active CPA metabolites [20], [21], DEAB appears to specifically inhibit ALDH1 [34]. Like hematopoiesis, intestinal epithelium is resilient following damage incurred during CPA therapy [25]. Hematopoietic stem cells (HSC) have high ALDH1 activity and can be isolated from bone marrow based on that unique trait [27], [28]. Similarly, we suggest that stem/progenitor cells in the base of colon crypts, like CoCSC, may have high ALDH1 activity, thus providing protection against CPA-induced cytotoxicity and tissue ablation during therapy. As *in vitro* experiments suggest, inhibition of ALDH activity *in vivo* sensitizes tumors to CPA therapy; however, the half-life of DEAB *in vivo* is extremely short [42] and such studies cannot be done. Knock down of *ALDH1A1* expression using a lentiviral-based shRNA approach in UM-C6 tumors demonstrated that CoCSC can be sensitized to CPA *in vivo*, as there was no noticeable enrichment of CoCSC in tumors from CPA- versus vehicle-treated mice. Furthermore, unlike for 4-HC *in vitro*, DEAB was unable to alter colorectal tumor cell sensitivity to Irinotecan, suggesting that the target of this inhibitor (ALDH1 enzyme activity) plays an important role in resistance to CPA, and resistance to Irinotecan appears to involve another mechanism.

Like DEAB, retinoic acid (a vitamin A/retinaldehyde metabolite and product of ALDH activity) appears to decrease ALDH1 and ALDH3 protein levels via a feedback mechanism that can sensitize cells to CPA-induced cytotoxicity [23], [43]. Retinoic acid, in the form of ATRA, is used with great success in the clinical setting for a subset of acute promyelocytic leukemia patients who have chromosomal translocations involving the retinoic acid receptor-α gene, *RARα* [47], but the use of retinoids in solid tumors has not been promising to date [48]. As we demonstrated here, either DEAB or ATRA alone sensitize colorectal tumor cells to 4-HC, and the combination of both appears synergistic. Because normal stem cell populations, such as HSC, neural stem cells and in all likelihood, intestinal stem cells, have high ALDH activity, its inhibition by ATRA as a pre-therapeutic regimen to CPA may also negatively impact normal stem cell populations. Nevertheless, as shown here, detailed study of rare tumor populations responsible for fueling tumor growth can provide mechanistic insights not only into tumorigenesis, but resistance mechanisms to common therapies. These inherent resistance mechanisms can include drug specific catabolic enzyme activity; such as that of ALDH1.

Here we show that xenogeneic colorectal tumors investigated to date contain a subset of TG ESA^+^CD44^+^ cells with high ALDH activity, and that this subpopulation is enriched in xenogeneic tumors from mice treated with CPA. These observations are supported by qRT-PCR using TG or NTG cells isolated by FACS, which show that *ALDH1A1* is the predominant cytoplasmic ALDH enzyme in colorectal tumors and its expression is further increased in residual tumor cells following therapy with CPA; consistent with the phenotypic increase in ALDH^+^ cells among the CoCSC phenotype. Importantly, however, ALDH1 activity alone does not confer tumorigenicity nor demarcate TG cells. When tumorigenicity of CD44^+^ versus CD44^−^ ESA^+^ALDH^+^ cells is compared, only the CD44^+^ subset is able to initiate actively growing tumors. Secondly, extended inhibition of ALDH1 activity with DEAB *in vitro* does not appear to alter cell proliferation or survival, as its presence in Irinotecan combination studies for 7 days *in vitro* did not differ from control. Furthermore, initiation of tumorigenesis with *ALDH1A1*- versus Luciferase-targeted shRNA containing cells was identical, demonstrating that ALDH1 enzymatic activity is not requisite in the absence of CPA exposure.

The advent of flow cytometry and cell sorting has revolutionized the study of developmental biology and disease, particularly in the hematopoietic system. Hematologic malignancies are among the best understood of the neoplastic diseases precisely because hematopoietic cells are easy to obtain and the *in vivo* and *in vitro* assays to determine the fate and potential of these cells have been developed. Similarly, the field of solid tumor biology has begun to enter an era where the cells responsible for fueling tumor growth can be identified, isolated, and their characteristics tested both *in vivo* and *in vitro*. Here we demonstrate for the first time that CoCSC are responsible for fueling both tumor growth and heterogeneity, and are enriched in residual tumors following chemotherapy. We also reveal that inherent resistance mechanisms differentially expressed within tumor subpopulations, such as ALDH1 enzymatic activity in CoCSC, can explain the inability of chemotherapeutic agents to improve overall survival despite tumor regression. In addition to providing evidence supported by serial transplantation studies for a previously unsupported tenet of the “cancer stem cell hypothesis”, we identify a major CSC-specific mechanism of resistance to a classical chemotherapeutic agent and establish experimental platforms both *in vitro* and *in vivo* for testing of novel agents either alone or in combination with standard of care therapies. Closer scrutiny of both normal tissue-resident stem cells and CSC will lead to a better understanding of disease mechanisms and, ultimately, better therapies.

## Materials and Methods

### Xenograft Line Propagation

Human colorectal tumor lines used in this study were obtained and passaged in mice as previously described [30]. Briefly, all tumors were initiated by subcutaneous implantation of either bulk tumor cells from frozen stocks or FACS purified cell populations into 6–8 week old NOD/SCID mice (Jackson Laboratories). Mice were anesthetized with Isoflurane or a single IP injection of 75–100 mg/kg Ketamine and 5–10 mg/kg Xylazine. Of the 3 xenograft lines used in this study, two (UM-C4 & UM-C6) originated at the University of Michigan and one (OMP-C8) originated at OncoMed Pharmaceuticals Inc. All experiments were carried out under approved institutional IACUC guidelines and protocols.

### Tissue Disaggregation and Cell Preparation

Tumor tissue was minced into tiny fragments (∼2 mm^3^), followed by enzymatic digestion with 300 u/mL Collagenase, 100u/mL Hyaluronidase, 0.5 mg/mL Dispase and 100 u/mL DNAseI (all obtained from Stem Cell Technologies; Vancouver, BC) for 1 hour at 37°C/5% CO_2_ with intermittent pipetting to disperse cells. Cells were then filtered sequentially through 70 µm and 40 µm screens, followed by a wash with excess FACS Buffer (1× Hanks Buffered Saline Solution [HBSS], 2% Heat-inactivated Fetal Calf Serum [FCS] and 25 mM HEPES [pH 7.4]). Red blood cells were lysed during a brief exposure to Ammonium Chloride and washed again with excess FACS buffer.

### Flow Cytometry and Cell Sorting

All analyses and cell isolations were performed using freshly dispersed cell suspensions. Antibody staining was performed in FACS buffer for 30 minutes at 4°C at a density of 1×10^7^ cells/mL. Antibodies used in this study include: anti-mouse H-2K^d^ (SF1-1.1; BD Pharmingen), anti-mouse CD45 (30-F11; BioLegend), anti-human ESA (HEA-125; Miltenyi Biotec), anti-mouse/human CD44 (IM7; eBioscience), anti-human CD49f (GoH3; BD Pharmingen) and anti-human CD166-PE (105902; R&D Systems). The Aldefluor™ reagent was purchased from StemCell Technologies and used per manufacturer instructions. In all experiments, cells staining positively for murine lineage markers (mLin^+^; H-2K^d^ and murine CD45) were excluded during flow cytometry using Cy5.5PE-labelled antibodies. Dead cells were excluded using the viability dye DAPI and cell doublets and clumps were excluded using doublet discrimination gating. Cellular phenotype and viability of FACS purified cells was confirmed by serial flow cytometric analysis prior to injection for tumorigenicity studies. Purity was typically >99%.

### Tumorigenicity Experiments

Following gentle centrifugation at 900 rpm×5 min, cells were resuspended in 50 µL of FACS buffer per mouse and mixed 1∶1 with Matrigel (BD Biosciences), followed by subcutaneous injection into the lower abdominal region while mice were under general anesthesia as described above. Only one tumor was initiated per mouse. Health was monitored daily and tumor growth was measured weekly using a digital caliper for up to 4 months. Animals were euthanized when tumors exceeded 1500 mm^3^ or the 120 day timepoint was reached. Statistical analysis of tumor growth includes only those mice with palpable tumors (Mean±SEM).

### Chemotherapy Regimen

Female mice were implanted with mLin^−^ESA^+^CD44^+^ cells (400 per mouse) while under anesthesia as described above. Once palpable, tumors were calipered twice weekly, and their length and width were used to calculate tumor volume based on the following formula: V = (length×[width])^2^/2. When tumors reached ∼400 mm^3^, mice were randomized into either the control group, which received vehicle (sterile water), or the therapeutic group, which received CPA administered intraperitoneally at the dose of 38 mg/Kg twice a week. Irinotecan was diluted in PBS and similarly administered once weekly at a dose of 15 mg/kg.

### Immunohistochemistry

Immunohistochemical studies were performed on formalin-fixed paraffin embedded tissues using monoclonal antibodies raised against Ki-67 (Vector Laboratories, catalog number VP-RM04) and c-Myc (DAKO, Catalog number M3570). Briefly, 4 µm-thick paraffin sections were deparaffinized and hydrated. Antigen retrieval was performed in 0.1M Tris-Cl, pH 9.0, using the Decloaking chamber from Biocare. Sections were then incubated with 3% hydrogen peroxide to block endogenous peroxidase activity and incubated with 1∶50 dilution of primary antibody for 1 hr at room temperature, and then detected using ImmPRESS anti-mouse or anti-rabbit Ig (peroxidase) followed by Vector® NovaRED™ substrate for visualization (Vector Laboratories). Hematoxylin was used for counter staining. TUNEL staining was performed using the In Situ Cell Death Detection™ kit (Roche). For Immunofluorescence staining of ALDH1 (Abcam), frozen sections were fixed with −20°C cold methanol for 10 min, followed by a blocking step. Sections were incubated with ALDH1 at 1 ug/ml for 1 hr at room temperature and visualized with anti-mouse antibody conjugated with Alexa Fluor 488. Anti-hapten IgG was used as negative control and mounted with Prolong Gold DAPI containing antifade (Invitrogen).

### *In Vitro* Culture & Cytotoxicity Studies

Following tissue disaggregation, as described above, cell suspensions were depleted of murine lineage cells using magnetic beads and plated in 96-well Primaria (BD Bioscience) plates at a density of ∼20,000 cells per well in serum free Medium-D (3∶1 low glucose DMEM:F-12 Media, B27 supplement, ITS-X, Pen/Strep [all from Invitrogen] and 0.5 µg/mL hydrocortisone [Stem Cell Technologies]), supplemented with 20 ng/mL bFGF and EGF, 5 u/mL Heparin and 1×10^6^ u/mL LIF. Plates were gently spun at 500 rpm for 5 min at room temperature following plating to promote attachment. When cultured *in vitro* for more than 7 days, media was changed weekly.

For cytotoxicity studies, cells were cultured for 3–4 days at 37°C/5% CO_2_/5% O_2_, non-attached cells were removed, and medium was replaced with that containing 4-hydroxycyclophosphamide (4-HC; obtained from Dr. OM Colvin; Duke University Medical Center), diethylaminobenzaldehyde (DEAB; Sigma), Irinotecan or their respective vehicle. In studies using 4-HC, cells were exposed for only 4 hours, after which the medium was removed, cells were washed twice with PBS, and fresh Medium-D was added. In experiments involving ATRA, cells were pre-incubated overnight prior to exposure to DEAB and/or 4-HC. Cell viability was then assessed using CellTitre-Glo (Promega) either 20 hr post-treatment for 4-HC studies, or 1 week after addition of Irinotecan.

### Lentiviral transduction of CoCSC

To assess clonality using lentiviral insertion site analysis, or introduce shRNA targeting ALDH1A1 or Luciferase, UM-C4 and UM-C6 tumor cells were transduced *in vitro* over a 3–4 day period. Lentiviral vectors used for these studies contained either an eGFP-Luciferase fusion gene (pLOM65) for clonality studies, or ALDH1A1 (pLOM205) or a control Luciferase (pLOM145) targeted shRNA embedded in the eGFP mRNA; each of which is driven by the CMV promoter. Cells were isolated by FACS based on GFP fluorescence and transplanted into mice. For clonality studies, pLOM65-transduced cells were deposited into 96-well plates in limiting dilution and single colonies were injected into mice 14 days later. Lentiviral insertion site analysis was performed as described elsewhere [49] using the *NlaIII* restriction endonuclease.

## Supporting Information

Figure S1 CoCSC phenotype cells preferentially survive CPA chemotherapy. UM-C6 tumors were initiated with ESA^+^CD44^+^ cells isolated by FACS. A) After randomization to normalize treatment groups at 400 mm^3^ at day 0, twice weekly administration of vehicle versus 38 mg/kg CPA commenced and UM-C6 tumors were measured periodically. B) Representative phenotypic analysis of vehicle-treated control and CPA-treated tumors for human UM-C4 ESA^+^CD44^+^CD166^+^ cells. Mean±SEM. C) Representative overlay histogram displaying the CD166 expression on human UM-C6 ESA^+^CD44^+^ cells.
(10.40 MB TIF)

Figure S2 Xenogeneic colorectal tumor lines contain a subset of ESA^+^CD44^+^ cells with high ALDH activity. Phenotypic profile of human ESA^+^ cells from various xenogeneic colorectal tumor lines for CD44 and ALDH enzymatic activity in the presence or absence of the ALDH1-specific inhibitor, DEAB.
(10.14 MB TIF)

Figure S3 UM-C6 CoCSC with high ALDH1 activity are more frequent following CPA therapy. Taqman qRT-PCR data for the denoted genes using TG and NTG populations from vehicle-treated (V) control or CPA-treated tumors. Data represents Mean±SEM (n≥2).
(6.61 MB TIF)
