# Correction: Colorectal Cancer Stem Cells Are Enriched in Xenogeneic Tumors Following Chemotherapy

**DOI:** 10.1371/annotation/2aa6a20a-e63c-49b6-aeea-aae62435617f

**Published:** 2008-08-21

**Authors:** Scott J. Dylla, Lucia Beviglia, In-Kyung Park, Cecile Chartier, Janak Raval, Lucy Ngan, Kellie Pickell, Jorge Aguilar, Sasha Lazetic, Stephanie Smith-Berdan, Michael F. Clarke, Tim Hoey, John Lewicki, Austin L. Gurney

In the Materials and Methods section, in the fifth line below the "*In Vitro* Culture & Cytotoxicity Studies" heading, the correct final concentration of LIF used in this study was 1 x 10e3 u/mL.

