# Supplementary figures and images for: Colorectal Cancer Stem Cells Are Enriched in Xenogeneic Tumors Following Chemotherapy
Source: PLoS One. 2008 Jun 18;3(6):e2428. doi: 10.1371/journal.pone.0002428 (PMC2413402; doi:10.1371/journal.pone.0002428)

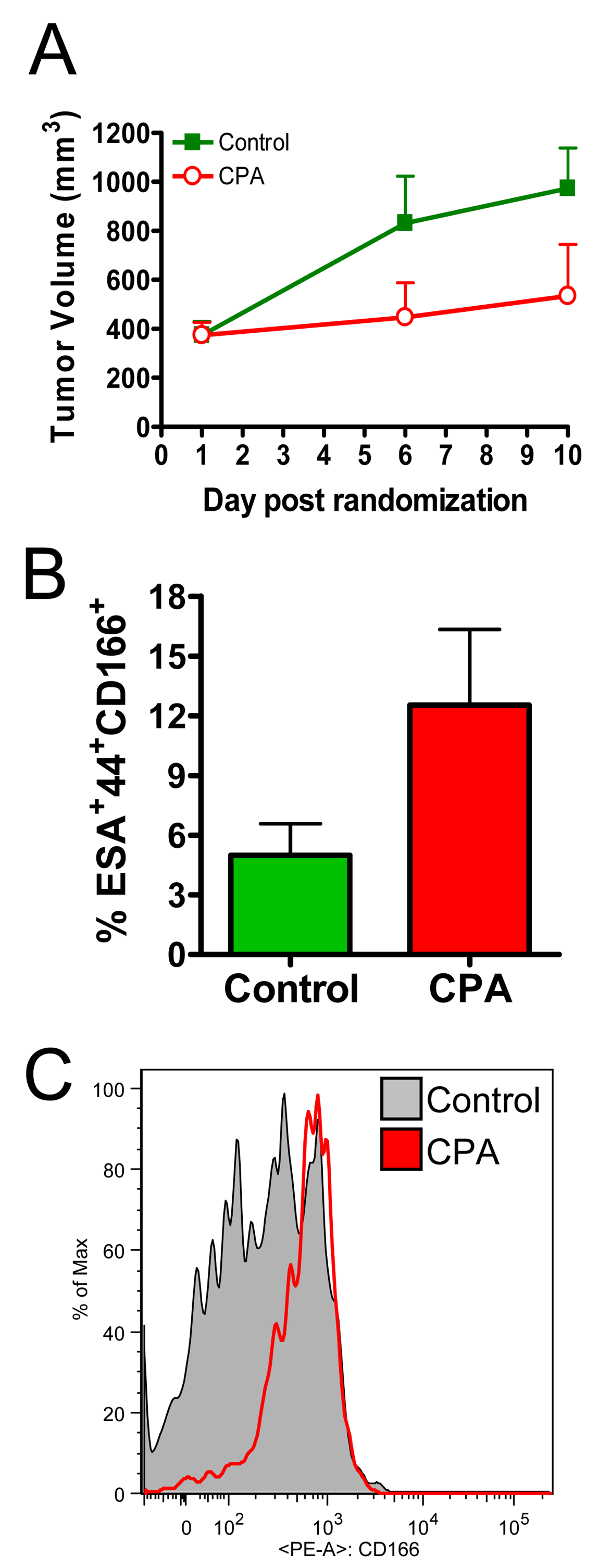

Supplement: Figure S1 — CoCSC phenotype cells preferentially survive CPA chemotherapy. UM-C6 tumors were initiated with ESA+CD44+ cells isolated by FACS. A) After randomization to normalize treatment groups at 400 mm3 at day 0, twice weekly administration of vehicle versus 38 mg/kg CPA commenced and UM-C6 tumors were measured periodically. B) Representative phenotypic analysis of vehicle-treated control and CPA-treated tumors for human UM-C4 ESA+CD44+CD166+ cells. Mean±SEM. C) Representative overlay histogram displaying the CD166 expression on human UM-C6 ESA+CD44+ cells. (10.40 MB TIF) [file pone.0002428.s001.tif]

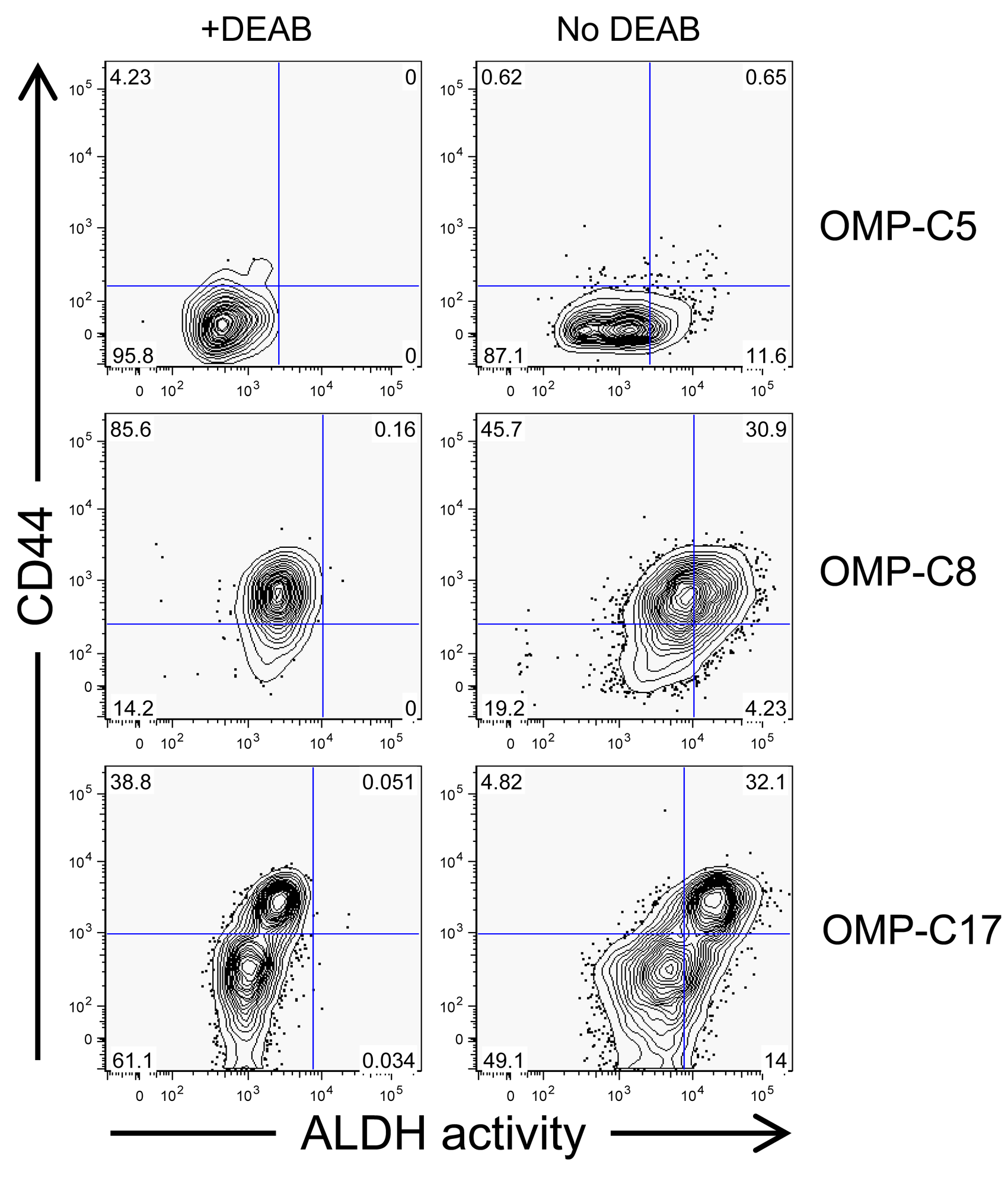

Supplement: Figure S2 — Xenogeneic colorectal tumor lines contain a subset of ESA+CD44+ cells with high ALDH activity. Phenotypic profile of human ESA+ cells from various xenogeneic colorectal tumor lines for CD44 and ALDH enzymatic activity in the presence or absence of the ALDH1-specific inhibitor, DEAB. (10.14 MB TIF) [file pone.0002428.s002.tif]

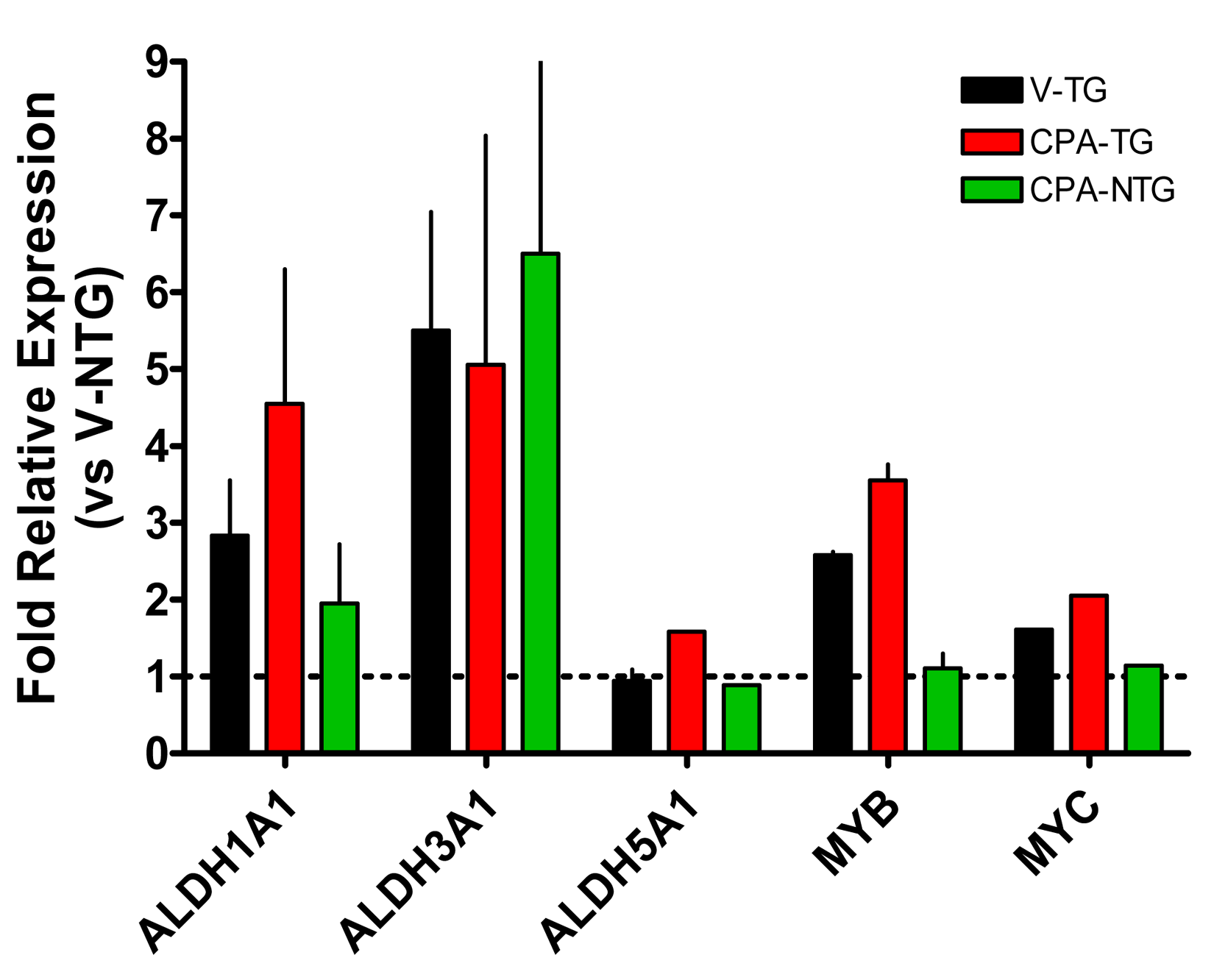

Supplement: Figure S3 — UM-C6 CoCSC with high ALDH1 activity are more frequent following CPA therapy. Taqman qRT-PCR data for the denoted genes using TG and NTG populations from vehicle-treated (V) control or CPA-treated tumors. Data represents Mean±SEM (n≥2). (6.61 MB TIF) [file pone.0002428.s003.tif]
